# Generative AI for medical 3D printing: a comparison of ChatGPT outputs to reference standard education

**DOI:** 10.1186/s41205-023-00186-8

**Published:** 2023-08-01

**Authors:** Aakanksha Sriwastwa, Prashanth Ravi, Andrew Emmert, Shivum Chokshi, Shayne Kondor, Kashish Dhal, Parimal Patel, Leonid L. Chepelev, Frank J. Rybicki, Rajul Gupta

**Affiliations:** 1 Department of Radiology, University of Cincinnati, College of Medicine, Cincinnati, OH 45219 USA; 2 Department of Orthopedics and Sports Medicine, University of Cincinnati, Cincinnati, OH 45209 USA; 3 Department of Mechanical and Aerospace Engineering, University of Texas, Arlington, TX 76010 USA; 4 Joint Department of Medical Imaging, University of Toronto, Toronto, Ontario, M5G 2N2 Canada; 5 Department of Biomedical Engineering, University of Cincinnati, College of Engineering and Applied Sciences, Cincinnati, OH 45219 USA

**Keywords:** Generative AI, Artificial intelligence, Radiology, 3D printing, Additive manufacturing, Rapid prototyping, Medical education, Postgraduate education

Generative AI Large Language Models (LLM) are designed to deliver coherent, constructive, and cogent output; they do so by generating the most statistically likely combination of words based on an input. There is great enthusiasm for Generative AI, including reports of how these technologies can improve medical education [1–3]. The models are “large” because they use extensive collections of text to build predictive models with billions to trillions of parameters [4–7]. OpenAI’s (OpenAI, San Francisco, CA, USA) ChatGPT (chat-based Generative Pretrained Transformer) and GPT-4 are potentially capable of bridging communication gaps and enhancing human interactions. These algorithms may hold promise in the medical sector.

ChatGPT incorporates a transformer neural network which uses sequential layers of attention and prediction fine-tuned using Reinforcement Learning from Human Feedback (RLHF) to generate responses to user prompts. GPT-3, from which ChatGPT was developed, had 96 such layers and 175 billion parameters [8]. It is specifically fine-tuned for conversational language understanding and generation. Simply put, it’s a chatbot trained on massive volumes of internet text [9]. Natural language processing enables it to deliver human-like text responses. It can process the user input, and models the output responses to the user input. Based on the human feedback, the favorable part of the output is reinforced, and the unfavorable part is edited to align with the user expectations [10]. GPT-based models have the potential to revolutionize the field of radiology and are being used increasingly for report generation, educational support, clinical decision support, patient communication and consent, and data analysis. As these models continue to improve, it is likely that more innovative uses for LLMs and GPT-based models in radiology will be developed, leading to benefits as well as challenges [11]. The accuracy of responses for ChatGPT was compared to Bard (Google LLC, Mountainview, CA, USA) and Bing (Microsoft Corporation, Redmond, WA, USA) when answering non-expert questions related to lung cancer. Although ChatGPT outputs had higher accuracy in comparison to other AI LLM outputs, none were always able to answer all the questions correctly [12, 13]. Further, LLMs have widely been used for improving readability and simplifying medical texts for people from non-medical backgrounds. Three LLMs (ChatGPT, GPT-4 and Google Bard) improved readability and reading ease for common patient questions regarding lung cancer and screening; Bard demonstrated the greatest improvement as well as preservation of the clinical appropriateness [13].

This Editorial applies ChatGPT to medical 3D printing, and it includes the benefits and limitations of the output. This editorial includes a series of queries from 3D printing program participants who have different levels and areas of expertise. Then, each query is evaluated with respect to a reference standard (Table 1). To our knowledge, Generative AI is previously untested for medical 3D printing, and, in particular for, 3D printing education. Nevertheless, providers and engineers are curious. 3D printing in Health Care Facilities (HCFs) is a new medical service, and there are limited if any multi-disciplinary training programs for providers. Educational opportunities exist for non-providers, including engineers and scientists, but the training slots only sometimes match educational needs. As the service moves from industry to HCFs, teaching in the hospital or affiliated university setting becomes more important, and provider education is needed for both the referring physicians as well as the physicians who are clinically responsible for the 3D printed parts. These stakeholders include the interventionalist (typically a surgeon), the 3D printing provider (often a radiologist), and one or more non-providers, including a biomedical engineer who performs and/or oversees the fabrication of the physical parts. Additionally, undergraduate and medical students add value and seek education in 3D printing labs. Without structured education, senior members of the group must spend substantial time teaching and mentoring them.

**Table 1:**
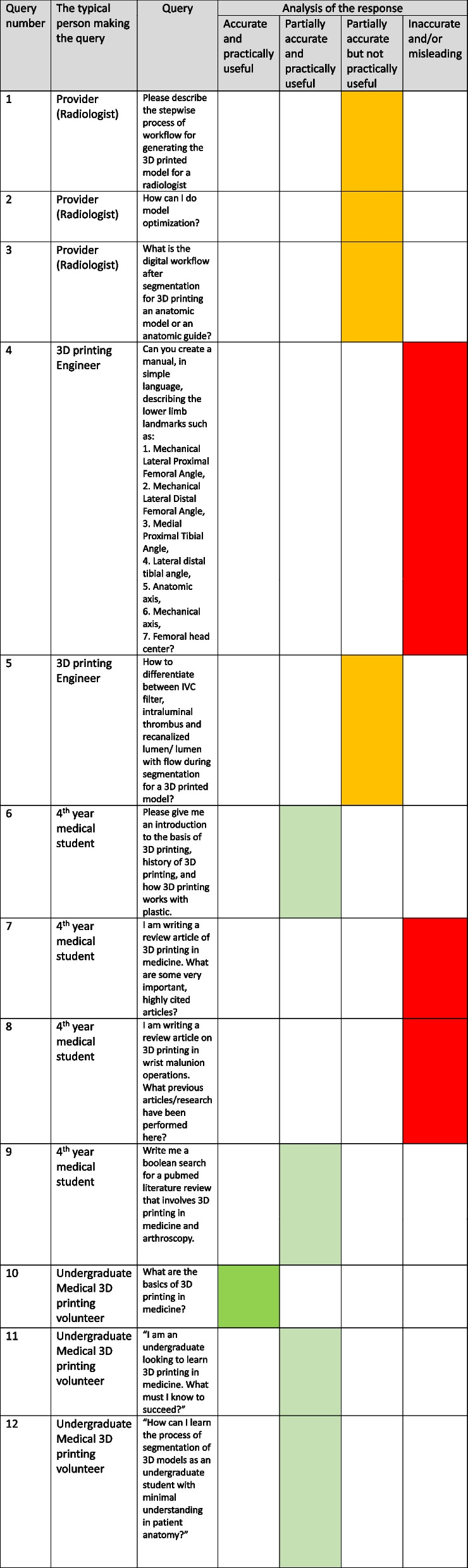
ChatGPT underwent 12 queries related to 3D printing; each query was analyzed with respect to a reference standard

The major educational challenge is that most stakeholders already undergo rigorous training programs that do not include 3D printing. There is little, if any, ‘elective’ time – for example, in a diagnostic radiology residency program, even though the fund of knowledge and practical experience needed to provide 3D printing as a clinical service is substantial. Success requires effective cross-disciplinary collaboration, and an automated strategy to incrementally provide education would be of great value. We hypothesized that Generative AI and ChatGPT, in particular, could potentially fill part of this educational void for 3D printing in HCFs. Admittedly, most of the ‘Methods’ presented are not scientifically rigorous. However, they practically editorialize the strengths and shortcomings of ChatGPT using different members of our clinical and research group.

## Query 1

### Typical person making the query and rationale

This query was from a radiologist (provider) who would become responsible for 3D printed parts. The query is motivated by shortcomings in 3D printing clinical training among physician providers. For example, other technologies (e.g., CT or MRI) are tested in the radiology professional board certification exams. Despite the benefits of the technology and the fact that programs are often centered in radiology, few radiology programs currently offer any formal education [10].

### Details of query and complete response

#### Query﻿

Please describe stepwise workflow for generating the 3D printed model for a radiologist.

#### Response

Sure, I'll be glad to outline a typical workflow for generating a 3D printed model for a radiologist. This involves converting medical imaging data into a 3D printable model. Here are the steps:

- Step 1: Data Acquisition

The first step in this process is the acquisition of the imaging data. This typically involves a computed tomography (CT) scan or a magnetic resonance imaging (MRI) scan.

- Step 2: Data Verification

Check the quality of the scan data to ensure it is suitable for 3D model generation. This ﻿might involve ensuring the scans have sufficient resolution, the area of interest is clearly visible, and the data is complete.

- Step 3: Image Segmentation

In this step, the region of interest (ROI) needs to be isolated from the rest of the image data. There are various software tools available that can assist with this process, including both commercial and open-source options. Some of these tools use machine learning algorithms to help automate the process, while others may require manual input.

- Step 4: 3D Reconstruction

Once the segmentation is complete, the next step is to use the segmented data to generate a 3D model. This process often involves creating a 3D mesh based on the segmented data. There are also software tools available that can help with this step, which can often be the same ones used for segmentation.

- Step 5: Model Optimization

The generated 3D model may need to be optimized before it can be printed. This can involve making sure the model is watertight (no holes), reducing the complexity of the model (decimation) if necessary, and adding support structures that will allow the model to be printed correctly.

- Step 6: 3D Printing

Once the model is prepared, it can be exported to a format that is compatible with a 3D printer, typically STL or OBJ file. The 3D printer then uses this file to print the model layer by layer, using a suitable material, often a type of plastic or resin for medical models.

- Step 7: Post-processing

After the 3D model has been printed, there may be some post-processing required. This can include removing any support structures used during printing, smoothing the surface of the model, and perhaps even painting the model to make certain features more visible.

- Step 8: Validation

The printed 3D model should be validated for accuracy. This step involves the radiologist or a medical expert, who checks the printed model against the original scan data to ensure it accurately represents the patient's anatomy.

- Step 9: Use in Practice

Now, the model is ready for use in medical practice. This could involve use in patient education, surgical planning, or even as a reference during a surgical procedure.

This process may vary depending on the specific needs of the radiologist and the patient, and the software and hardware being used. It's always best to consult with the necessary professionals throughout the process to ensure the model is accurate and fit for purpose.

### Reference standard

Reference standard was a discussion with a 3D printing engineer.

### Analysis of the response

When asked about the stepwise workflow for generating the 3D printed model, ChatGPT appropriately enumerated the following steps: data acquisition and verification, image segmentation, 3D reconstruction based on segmented data, model optimization, 3D printing, post-processing, validation, and finally use in practice. On further prompting, the software elaborated on each in detail. (For brevity, these additional queries and responses are not shown.) However, all the outputs invariably came with a disclaimer to consult with the necessary professionals throughout the process to ensure accuracy and fit of the model.

## Query 2

### Typical person making the query and rationale

This query was also from a radiologist, who asked ChatGPT about model optimization. The rationale for this query was that radiologists lack formal training in the properties of surface mesh files and how to manipulate them in the 3D printing workflow.

### Details of the query and complete response

#### Query

How can I do model optimization?

#### Response

Model optimization for 3D printing is an essential step in the preparation of a 3D model for printing. Depending on the model's complexity and the type of 3D printer being used, different types of optimizations may be necessary.

Here are some basic steps to optimize a 3D model for printing:

- 1. Ensure the model is Watertight

This means that the model should not have any holes or gaps in it. Any missing faces or edges can cause problems during the 3D printing process. Software such as Meshmixer, Netfabb, or MeshLab can be used to identify and fix any holes or other issues.

- 2. Simplify the Model if Necessary

This is also known as decimation. Sometimes, the 3D model may have more detail than the 3D printer can accurately produce, or more detail than is necessary for the purpose of the model. In these cases, reducing the model's complexity can make it easier to print without significantly impacting the model's utility. Tools for this can also be found in the aforementioned software.

- 3. Check for Overhangs

Overhangs are parts of the model that stick out, often horizontally, without anything underneath them. Most 3D printers print from the bottom up and can struggle with overhangs that are too steep. If the overhangs are too extreme, you might need to add support structures, or consider splitting the model and then assembling it post-print.

- 4. Add Support Structures if Necessary

As mentioned above, support structures may be needed for models with overhangs or complex geometries. Many slicing software, which prepare the 3D model for the printer, include automatic support generation, but manual adjustment may sometimes be necessary.

- 5. Ensure Correct Orientation

The model's orientation in the printer can affect the success of the print. The orientation can impact the strength of the printed object, the amount of support needed, and the appearance of the final print. You can adjust the orientation of the model in the slicing software.

- 6. Scale the Model Appropriately

Ensure that the model is correctly scaled for your needs. The model may need to be scaled down to fit the printer's build volume, or you may wish to scale the model up or down for other reasons.

Remember, always preview your model in your slicing software before you print. This allows you to see a layer-by-layer representation of the print, and you can spot potential issues before they become real problems.

### Reference standard for query 2

Reference standard was a discussion with a 3D printing engineer.

### Analysis of query 2

There were no factual errors in the ChatGPT output. However, when the output was compared to expert reference standard, shortcomings were related to the lack of detail and refinement of the ChatGPT response. Specifically, ChatGPT did not include specific details related to actual cases. For example, a part may not be water-tight. [14]. Overhangs with a distant apex and more than 45° are unsupported and tend to break when taken off the tray [15]. The ChatGPT reply lacked experience-based tools, and particular examples from specific cases. Thus, while the response was factually correct, it did not have the scope of knowledge that would be expected to train the radiologist.

## Query 3

### Typical person making the query and rationale

This query was also from the radiologist, and it concerned the digital workflow after image segmentation that typically occurs in the DICOM space. Radiologists are experts in anatomy and image segmentation. However, radiologists seldom if ever work outside of DICOM data sets. Thus, Computer Aided Design (CAD) and the file types (e.g., STL flies) are largely unknown. At the onset of 3D printing in a HCF, a radiologist is likely to utilize free software such as DEP Meshworks® (Detroit Engineering Products, Troy, MI, USA) or 3D Slicer (www.slicer.org) [16].

### Details of the query and complete response

#### Query

What is the digital workflow after segmentation for 3D printing an anatomic model or an anatomic guide?

#### Response

After segmentation, the digital workflow for 3D printing an anatomical model or guide typically involves several steps. Here's a general outline of the process:

- Segmentation: This is the initial step where medical imaging data, such as CT or MRI scans, are processed using specialized software to isolate the anatomical structures of interest. Segmentation allows for the extraction of the desired region or organ from the overall scan.
- 3D Model Creation: Once the segmentation is complete, the segmented data is used to create a 3D model. This is achieved by converting the segmented data into a digital representation of the anatomical structure using medical imaging software or specialized 3D modeling software.
- Model Refinement: The generated 3D model may require refinement to remove any artifacts, smooth the surfaces, or enhance the details. Various tools and software can be used for this purpose, including mesh editing tools or sculpting software.
- Model Preparation: The refined 3D model needs to be prepared for 3D printing. This involves processes such as mesh repair, orientation adjustment, and support structure generation. Mesh repair fixes any issues with the model's geometry, while orientation adjustment ensures optimal printing position. Support structures are generated to provide stability during the printing process.
- Slicing: The prepared 3D model is then imported into slicing software, which divides the model into thin, printable layers. The software determines parameters like layer thickness, infill density, and printing speed. Slicing software generates a set of instructions, typically in the form of G-code, which the 3D printer understands.
- Printing: The sliced model is transferred to a 3D printer capable of printing with appropriate materials, such as biocompatible resins or filaments. The 3D printer follows the instructions from the slicing software to deposit and cure the material layer by layer, gradually building the physical model.
- Post-Processing: Once the printing is complete, the 3D printed model may require post-processing steps. This can involve removing support structures, sanding or polishing the surface for better aesthetics, and applying any necessary finishing touches.
- Validation and Quality Control: The printed model should be inspected for accuracy and quality. It can be compared to the original medical images or evaluated by medical professionals to ensure it accurately represents the anatomical structure of interest.

It's worth noting that the specific tools, software, and techniques used in the workflow may vary depending on the software preferences, hardware capabilities, and the intended application of the 3D printed anatomical model or guide.

### Reference standard

Reference standard was a discussion with a 3D printing engineer.

### Analysis of the response

ChatGPT’s response to this query was factually correct. However, once again the output lacked details and in-depth information. It also lacked the practical tips essential for application of 3D printing technology. ChatGPT failed to mention the precautions that need to be taken while printing a model with thin structures (like blood vessels or unsupported/ weakly supported ribs) concerning the model’s orientation. The 3D printing specialist highlighted that this is one of the commonly encountered potential challenges in practical 3D printing and post-processing of the physical parts. The thinner structures might break in the post-processing phase if the supports are directly attached to them. Therefore, the model orientation should ensure no/minimal support pillars attaching to these fragile structures, or they can be reinforced with connecting pins, instead of pillars while printing. The possible correct troubleshooting solution was only provided after inputting multiple lead questions, which is hardly feasible in real life, especially when one is unaware of the full scope of one’s knowledge gaps.

Commercially available online search engines may do the same task as ChatGPT. An important and rather impactful difference, however, is that ChatGPT summarizes all the relevant information into an easy paragraph with bullet points for ease of comprehension. Instead of juggling multiple tabs and sifting through a sea of articles to find the most pertinent and up-to-date information on a given topic, as one would typically do with Google or other web browsers, ChatGPT streamlines this process by delivering a succinct, easily digestible summary. It parses the vast web of information, curating the most relevant content and translating it into a language that's more accessible and comprehensible to the user. However, as realized in this query, the downside is that the outputs are not detail-oriented and thus have real limitations for common 3D printing practice. An additional downside is that the provided outputs may not be factually correct, and there is limited capability of an individual with a restricted understanding of a particular specialist field to assess whether a specific statement is fabricated or true.

## Query 4

*Typical person making the query and rationale:* This query was made by an orthopedic surgeon. This query relates to anatomic models and anatomic guides from a referring provider orthopedic surgeon. The surgeon requests patient-specific 3D printing in an HCF. Correction of bony deformities requires that everyone within the care team be well-informed about the surgical plan. Collaboration with the 3D printing team is essential for pre-operative planning. This interaction requires substantial time investment, and there are risks for communication errors among the team members. Without direct interactions with the surgeon, the 3D printing engineer would otherwise have limited access to the information in this query.

The rationale for the detail in this query was to evaluate the accuracy of ChatGPT to match established lower limb axes and angles. When presented with a patient needing a corrective osteotomy or a soft tissue surgery, understanding these angles and relationships is a pre-requisite to arrive at the final anatomic representation that, in turn, would be 3D printed. The questions focus on the lower (as opposed to the upper) limbs since they represent the most encountered clinical scenarios that requires 3D printing.

### Details of the query and complete response

#### Query

Can you create a manual, in simple language, describing the lower limb landmarks such as:

1. Mechanical lateral proximal Femoral angle
2. Mechanical lateral distal Femoral angle
3. Medial proximal tibial angle
4. Lateral distal tibial angle
5. Anatomic axis
6. Mechanical axis
7. Femoral head center?

#### Response

Certainly. Here is a step-by-step guide to measure the limb angles on X-ray images with all the line in the same plane.

1. Mechanical Lateral Proximal Femoral Angle (mLPFA)

On X-ray images, follow these steps:

- Identify the center of Femoral head and neck and the center of Femoral shaft.
- Draw a line through the center of Femoral head and neck.
- Measure the angle between these two lines using a goniometer.

2. Mechanical Lateral Distal Femoral Angle (mLDFA)

To measure the mLDFA on X-ray images, follow these steps:

- Identify the center of femoral shaft and the center of tibial plateau.
- Draw a line through the center of femoral shaft, and a second line through the center of tibial plateau.
- Measure the angle between these two lines using a goniometer.

3. Medial Proximal Tibial Angle (MPTA)

To measure the MPTA on an X-ray image, follow these steps:

- Identify the center of tibial plateau and the ankle joint.
- Draw a line through the center of tibial plateau.
- Draw a second line through the tibial shaft.
- Measure the angle between these two lines using a goniometer.

4. Mechanical Lateral Distal Tibial Angle (LDTA):

To measure the LDTA on an X-ray image, follow these steps:

- Identify the center of the Tibial plateau and the ankle joint.
- Draw a line through the center of Tibial plateau.
- Draw a second line through the ankle joint.
- Measure the angle between these two lines using a goniometer.

5. Anatomical axis:

To determine the anatomical axis on an X-ray image, follow these steps:

- Identify the center of the Femoral head and neck, the center of the knee joint, and the center of ankle joint. Draw a line through the center of Femoral head and neck.
- Draw a second line through the center of knee joint and the center of ankle joint.
- Measure the angle between these two lines.

6. Mechanical axis:

To determine the mechanical axis on an X-ray image, follow these steps:

- Identify the center of the hip joint, the center of the knee joint, and the center of the ankle joint.
- Draw a line through the center of hip joint and the center of knee joint.
- Draw a second line through the center of knee joint and the center of ankle joint.
- Measure the angle between these two lines.

7. Femoral head center:

To determine the Femoral head center on X-ray images, follow these steps:

- Identify the center of the Femoral neck and the center of the femoral head.
- Draw a line from the center of Femoral neck to the center of femoral head.
- Measure the distance between center of Femoral head and the line you draw.
- Again, it’s important to note that these lines should be in the same plane.

### Reference standard for query 4

The Reference standard was a discussion with an orthopedic surgeon.

### Analysis of the response

**Fig. 1:**
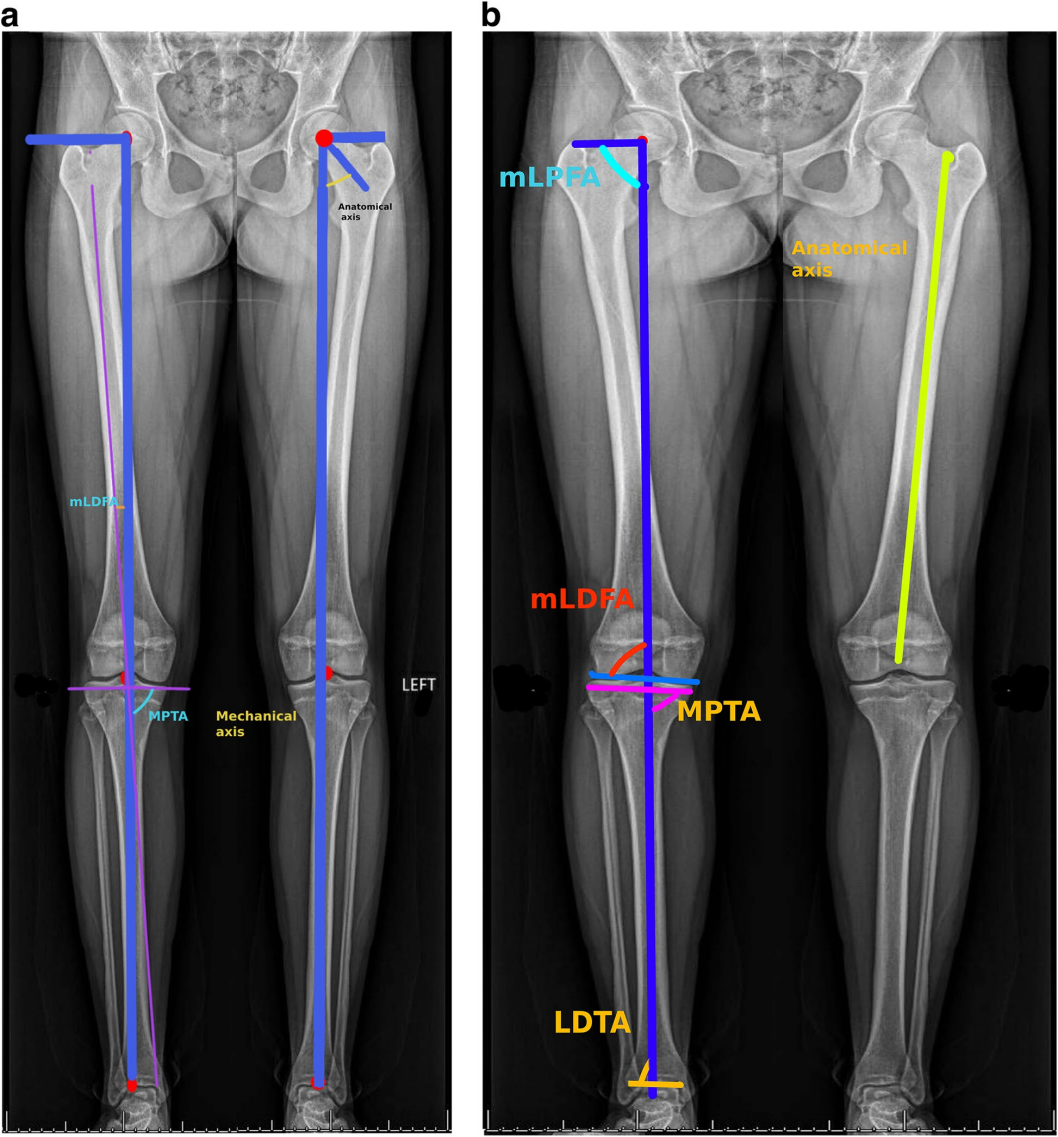
**A** Lower extremity landmarks, angles and axes as described by ChatGPT. The output of ChatGPT was illustrated on standing radiographs of the lower extremities. **B** Reference standard landmarks, angles and axes illustrated by an orthopedic surgeon. mLPFA – mechanical Lateral Proximal Femoral Angle. mLDFA – mechanical Lateral Distal Femoral Angle. MPTA – Medial Proximal Tibial Angle. LDTA – Lateral Distal Tibial Angle

The ChatGPT output was interpreted by the reference standard orthopedic surgeon who then translated the ChatGPT output into a radiographic illustration of the angles and axes of the lower extremity (Fig. 1A) along with the same illustration that would be standard in clinical practice (Fig. 1B). Table 2 compares ChatGPT’s output with the standard method of identifying the required angles and axes.

**Table 2:** Comparison between reference standard methods used in everyday orthopedic clinical practice and ChatGPT. For each numbered angle or axis, clinical importance is provided along with reference standard methods to mark axes and angles of the lower limbs. The analysis of the ChatGPT output includes the reasons why the ChatGPT output is accurate or inaccurate

	**Angle/Axis**	**Reference Standard**	**ChatGPT**	**Analysis**
1.	Center of femoral head *The entire lower limb rotates around this very important landmark. The center of the femoral head is also the center of the hip joint.*	Draw the best fitting circle over the femoral head. The location of the femoral head is defined by this circle and its center.	Identify the center of the femoral neck and the center of the femoral head. Draw a line from the center of the femoral neck to the center of the femoral head. Measure the distance between the center of the femoral head and the line you drew.	ChatGPT is entirely wrong. *The center of the femoral head is a single point. Since the normal femoral head is almost spherical, this point becomes an essential landmark. ChatGPT defines and suggests drawing a line.*
2.	Mechanical axis *The mechanical axis is a line from the center of the femoral head to the center of the ankle joint; it is the direction of the force vector for weight bearing. Disturbances of mechanical axis are very undesirable because the abnormal forces will be distributed on the lower limb bones and joints.*	Mechanical axis is a straight line between center of the femoral head and the center of the ankle joint.	Identify the center of the hip joint, the center of the knee joint, and the center of the ankle joint. Draw a line through the center of the hip joint and the center of the knee joint. Draw a second line through the center of the knee joint and the center of the ankle joint. Measure the angle between these two lines.	ChatGPT is partially correct. *While the mechanical axis does pass through the center of hip and ankle joint, it does not pass through the center of knee joint if there is any bone or joint deformity. Therefore, Chat GPT is only partially correct.*
3	Anatomical axis *These axes are the geometric long axes of bone. The normal difference between the anatomical and mechanical axis of the femur is about 6 degrees. This relationship is disturbed by fractures or deformities. One goal of surgery is to restore these angular relationships.*	Anatomical axis is the axis of the femoral shaft and the tibial shaft. In femur, there is a difference between anatomical and mechanical axis of a few degrees, but there is no difference among these axes in tibia.	Identify the center of the femoral head and neck, the center of the knee joint, and the center of the ankle joint. Draw a line through the center of the femoral head and neck. Draw a second line through the center of the knee joint and the center of the ankle joint. Measure the angle between these two lines.	ChatGPT is entirely wrong. *There are two problems. First, anatomic axis does not involve the femoral neck, as ChatGPT describes. Second, ChatGPT instructs how to draw an angle. This is not relevant.*
4	Mechanical Lateral Proximal Femoral Angle (mLPFA) *This angle is used to define the bony deformity of the femur.*	Draw a horizontal line between the center of the femoral head and the mechanical axis on the lateral aspect. Draw Mechanical axis. The angle is between the two lines	Identify the center of the femoral head and neck and the center of the femoral shaft. Draw a line through the center of the femoral head and neck. Draw a second line through the center of the femoral shaft. Measure the angle between these two lines using a goniometer.	ChatGPT is entirely wrong. It incorrectly provides instruction regarding how to draw a neck shaft angle. *The angle between femoral neck and shaft is called neck shaft angle; it is completely different than the mLPFA.*
5	Mechanical Lateral Distal Femoral Angle (mLDFA) *This angle is used to characterize deformities of the knee and the inferior part of femur.*	Draw Mechanical axis. Draw a line between the distal ends of medial and lateral femoral condyles. mLDFA is the angle between the two lines.	Identify the center of the femoral shaft and the center of the tibial plateau. Draw a line through the center of the femoral shaft. Draw a second line through the center of the tibial plateau. Measure the angle between these two lines using a goniometer.	ChatGPT is entirely wrong. *The tibial plateau (the upper broad part of tibia) is not used to determine the mLDFA.*
6	Medial Proximal Tibial Angle (MPTA) *This angle is used to characterize deformities of the knee and the superior part of the tibia.*	Draw mechanical axis. Draw a line parallel to the articular surface of tibia. The angle between the two lines in MPTA.	Identify the center of the tibial plateau and the tibial shaft. Draw a line through the center of the tibial plateau. Draw a second line through the tibial shaft. Measure the angle between these two lines using a goniometer	ChatGPT is entirely wrong. *The horizontal line must be parallel to the surface of the upper end of tibia. ChatGPT is inaccurately describing the angle.*
7	Lateral Distal Tibial Angle (LDTA) *This angle is used to characterize a deformity near the inferior tibia and the ankle joint.*	Draw the mechanical axis. Draw a line parallel to distal tibial articular surface. The angle between the two lines is LDTA.	Identify the center of the tibial plateau and the ankle joint. Draw a line through the center of the tibial plateau. Draw a second line through the ankle joint. Measure the angle between these two lines using a goniometer.	ChatGPT is entirely wrong. *ChatGPT fails to mention the vertical line that is the mechanical axis of tibia used to determine LDTA.*

The ChatGPT output misrepresented operative planning and therefore would not be effective for 3D printing education. For this query, it does not simplify the process of communication between the surgical team and the 3D printing team. Its failure to reproduce widely available and long-established methods is misleading. For the 3D printing engineer who otherwise would not be familiar with the nuances of lower limb deformity correction, this output would be potentially harmful.

## Query 5

### Typical person making the query and rationale

This query was from a 3D printing engineer. Providers from multiple specialties in an academic medical center tend to request 3D printed models for more complex cases. This exposes a knowledge gap between the clinicians and the 3D printing engineers. In another practical example, an interventional radiologist requests a 3D printed model of an Inferior Vena Cava (IVC) filter that is holding thrombus, demonstrated on a contrast enhanced CT scan. Segmenting the various Regions of Interest (ROIs) that represent the relevant anatomy can be challenging for a non-radiologist. Accurate differentiation of partially thrombosed IVC lumen from the areas of flow/ partial recanalization was technically difficult for the 3D printing engineer. ChatGPT can be potentially used to rapidly fill this knowledge gap.

### Details of the query and complete response

#### Query

How to differentiate between IVC filter, intraluminal thrombus and recanalized lumen/ lumen with flow during segmentation for a 3D printed model?

#### Response

To differentiate between an IVC filter, intraluminal thrombus, and a recanalized lumen or lumen with flow during segmentation for a 3D printed model, you can consider the following approaches:

- Visual Appearance: Analyze the visual appearance of the structures on the contrast-enhanced CT images. An IVC filter will typically have a distinct metallic appearance with radiopaque struts, allowing it to be easily differentiated from other structures. Intraluminal thrombus may appear as irregular filling defects within the vessel lumen, while a recanalized lumen or lumen with flow will typically show continuous contrast enhancement along the vessel course.
- Hounsfield Unit (HU) Values: Utilize the Hounsfield Unit values derived from the CT images to aid in differentiation. An IVC filter will exhibit HU values indicative of the metallic material used in its construction. Intraluminal thrombus may have variable HU values depending on its composition and age, ranging from hyperdense to isodense with blood. A recanalized lumen or lumen with flow will typically have HU values similar to that of blood.
- Shape and Location: Consider the shape and location of the structures within the vessel. An IVC filter will have a specific shape and position within the IVC, often spanning across its diameter. Intraluminal thrombus may appear as irregular filling defects conforming to the vessel's contours, while a recanalized lumen or lumen with flow will typically exhibit a continuous tubular shape along the vessel course.
- Clinical Context: Take into account the clinical context and patient history. If the patient has a known history of IVC filter placement, the presence of a metallic structure within the IVC strongly suggests an IVC filter. Additionally, clinical information regarding the presence of thrombotic events, the presence of a previously occluded vessel, or evidence of recanalization can help guide the interpretation of the CT images.

Segmentation of these structures may involve employing various image processing techniques, such as thresholding, region growing, or edge detection algorithms. Additionally, consulting with a radiologist or a medical imaging expert can provide valuable insights and guidance in accurately identifying and segmenting these structures based on the specific CT images and clinical context.

### Reference standard

The Reference standard was a discussion with a radiologist managing a 3D printing clinical service line in an HCF.

### Analysis of the response

The response was accurate and provided useful information about the IVC filter and the varying attenuation values. The output had practical value but was not sufficient to replace the experience that the radiologist provided for image segmentation of the ROIs. For example, there is low attenuation artifact (beam hardening) that is adjacent to the metallic struts. In some cases, this artifact can be readily recognized by an engineer because it extends outside the confines of the IVC. However, in several cases the artifact is difficult to separate from fibrin that is adherent to the struts or thrombus. Thus, while the 3D printing engineer benefited, ChatGPT was unable to supplant the radiologist’s final segmentation and approval of the images to generate the final anatomical representation.

## Query 6

### Typical person making the query and rationale

This query was from a fourth-year medical student. Several medical students incorporate 3D printing into their education, with an increasing number of printers in medical school libraries. However, most medical students receive no formal education in 3D printing during their pre-clinical or clinical training [17, 18]. Medical students seek lab experience, and then supplement this with self-guided introductory modules to medical 3D printing. Several resources are available online and are freely accessible [19]. However, the terminology and layout of such 3D printing guides can easily confuse a medical student with no background in biomedical engineering or other fields related to 3D printing. The query was designed to challenge the algorithm’s ability to simplify medical 3D printing introductory modules.

### Details of the query and complete response

#### Query

Please give me an introduction to the basics of 3D printing, history of 3D printing, and how 3D printing works with plastic.

#### Response

Sure! 3D printing, also known as additive manufacturing, is a technology that allows for the creation of three-dimensional objects by layering materials on top of each other. The process starts with a digital 3D model, which is then sliced into thin layers by specialized software. These layers are then printed one at a time, building up the final object.

The history of 3D printing dates back to the 1980s, when the first patent for the technology was filed by Chuck Hull, who invented a process called stereolithography. This early method involved using lasers to solidify layers of liquid plastic, and it paved the way for other forms of 3D printing that followed.

Over the years, 3D printing technology has advanced significantly, and today it is used in a wide range of industries, from aerospace and automotive to healthcare and consumer products.

When it comes to 3D printing with plastic, there are several different methods that can be used, but the most common is fused deposition modeling (FDM). In FDM, a spool of thermoplastic filament is fed into a printer head, which heats the material until it melts, the printer head then moves along a set path, laying down the melted plastic in thin layers to create the object.

FDM is a relatively simple and affordable method of 3D printing, which is why it has become so popular. However, there are other methods, such as stereolithography and selective laser sintering, which can be used to print with other materials, such as resins and metals.

### Reference standard

The reference standard was “Ultimate Guide To: Medical 3D Printing Applications”(Sculpteo 3D Printing Learning Hub) [20].

### Analysis of the response

Based on the simplicity of the request, the output from ChatGPT was considered to have some practical use (Table 1). The query had a textual reference standard, and ChatGPT's output was generally more difficult to read (Table 3). The query did not include medical applications, but based on the query and output, it was considered accurate and useful.

**Table 3:** Comparison of readability metrics of introductory modules to medical 3D printing generated by humans and ChatGPT [21]

**Metric**	**Human**	**ChatGPT**
Flesch Reading Ease score	50.5 (fairly difficult to read)	51.5 (fairly difficult to read)
Gunning Fog	12.4 (hard to read)	13.5 (hard to read)
Flesch-Kincaid Grade Level	10.2 (tenth grade)	11.3 (eleventh grade)
The Coleman-Liau Index	11 (eleventh grade)	10 (tenth grade)
The SMOG Index	9.5 (tenth grade)	9.5 (tenth grade)
Automated Readability Index	9.4 (eighth and ninth grade)	11.5 (twelfth grade)
Linsear Write Formula	10.7 (eleventh grade)	13.3 (college)

## Query 7

### Typical person making the query and rationale:

This query was from a fourth-year medical student.

### Details of the query and complete response

#### Query

I am writing a review article on 3D printing in medicine. What are some very important, highly cited articles?

#### Response:

1. “Three-dimensional printing in medicine" by Tack et al. (2016) - This article provides an overview of the different 3D printing technologies and materials used in medicine and summarizes the applications of 3D printing in various medical fields.
2. “Medical applications of 3D printing: a review" by Wong et al. (2016) - This review article examines the use of 3D printing in medicine and discusses the various types of medical applications, including surgical planning, implant and prosthesis design, and drug delivery systems.
3. “The clinical application of 3D printing in orthopedic surgery" by Li et al. (2019) - This article focuses on the use of 3D printing in orthopedic surgery, including the design and fabrication of custom implants, surgical guides, and anatomical models for preoperative planning.
4. "Three-dimensional printing and its applications in otorhinolaryngology-head and neck surgery" by Zhang et al. (2018) - This review article highlights the use of 3D printing in otorhinolaryngology-head and neck surgery, including the production of patient-specific implants and models for surgical planning and education.
5. "3D printing in cardiovascular medicine" by Vukicevic et al. (2018) - This article examines the use of 3D printing in cardiovascular medicine, including the production of models for surgical planning, the development of patient-specific devices, and the fabrication of vascular grafts.

### Reference standard

The reference standard was a Pubmed (https://pubmed.ncbi.nlm.nih.gov/) search.

### Analysis of the complete response

ChatGPT output was cogent, but not necessarily true. This phenomenon is called large language model hallucination and can present as complete fabrication [22]. The first listed publication exists under the title “3D-printing techniques in a medical setting: a systematic literature review” [23] instead of “Three-dimensional printing in medicine” as listed by ChatGPT. The author as well as the year are accurate. The second listed publication has an incorrect citation, however, there is an article written by KC Wong as a sole author in 2016 titled “3D-printed patient-specific applications in orthopedics” [24]. The third listed publication does not exist under the specific title or year. However, there is an article titled “Three-dimensional printing: The potential technology widely used in medical fields” written by Li et al. in 2020 [25]. The fourth listed publication has an incorrect author and year. The article was written by Crafts et al. in 2017 [26]. The fifth listed publication has an accurate year and author, however the title exists as “Cardiac 3D printing and its future directions” [27].

It appears that ChatGPT properly identified the general features of some of the highly cited articles in the field. However, it provided inaccurate citations for each of the articles it found. The results of this query led to the following, more focused query.

## Query 8

### Typical person making the query and rationale

This query was from a fourth-year medical student.

### Details of the query and complete response

#### Query

I am writing a review article on 3D printing in wrist malunion operations. What previous articles/research has been performed here?

#### Response:

1. "3D printing in wrist fractures and post-traumatic deformities: a review of the literature" by Yew et al. (2020) - This review article provides an overview of the applications of 3D printing in wrist fractures and post-traumatic deformities, including the use of 3D printed models for preoperative planning, the design and production of custom-made plates and screws, and the fabrication of patient-specific implants.
2. "Surgical treatment of malunion of the distal radius with 3D-printed osteotomy templates: a case report" by Tan et al. (2017) - This case report describes the use of 3D-printed osteotomy templates in the surgical treatment of a malunion of the distal radius. The authors demonstrate how 3D printing technology can be used to produce precise and patient-specific guides for osteotomies.
3. "Three-dimensional printing in the surgical treatment of traumatic fractures of the distal radius" by Wang et al. (2019) - This study investigates the use of 3D printing in the surgical treatment of traumatic fractures of the distal radius. The authors report that 3D printing technology can help improve the accuracy of osteotomies, reduce the risk of complications, and enhance postoperative functional outcomes.
4. "Custom-made 3D-printed osteotomy template for the treatment of malunion of the distal radius: a case report" by Tsai et al. (2019) - This case report describes the use of a custom-made 3D-printed osteotomy template in the treatment of a malunion of the distal radius. The authors demonstrate how 3D printing technology can help produce accurate and patient-specific guides for corrective osteotomies.
5. "3D-printed patient-specific surgical plates for the treatment of distal radius fractures: a prospective pilot study" by Wu et al. (2021) - This pilot study investigates the use of 3D-printed patient-specific surgical plates in the treatment of distal radius fractures. The authors report that the use of 3D printing technology can help produce plates that fit better and provide more stable fixation compared to off-the-shelf implants.

### Reference standard

As in the 7^th^ query, the reference standard was a Pubmed (https://pubmed.ncbi.nlm.nih.gov/) search.

### Analysis of the response

To the authors’ knowledge, no article exists for the first listed publication. Similarly, no article exists in terms of wrist malunions and 3D printing that involves the listed author, listed year, or listed title for the second and third publication. An article with the title of the fourth article does not exists, however, there is an article titled “3D-printed flexible polymer stents for potential applications in inoperable esophageal malignancies” with Tsai as the third author, published in 2019 [28]. Additionally, the fifth listed publication does not exist. However, there is an article published in 2018 unrelated to wrist malunions titled “Three-dimensional printing of patient-specific surgical plates in head and neck reconstruction: A prospective pilot study,” which is similar to the title listed by ChatGPT [29]. These suboptimal responses prompted the next query.

## Query 9

### Typical person making the query and rationale:

 This query was from a 4^th^year medical student.

### Details of the query and complete response:

#### Query

Write me a boolean search for a PubMed literature review that involves 3D printing in medicine and arthroscopy.

#### Response

(("3D printing" OR "additive manufacturing" OR "three-dimensional printing") AND ("arthroscopy" OR "joint surgery") AND ("orthopedics" OR "musculoskeletal system" OR "bone fractures" OR "soft tissue injuries" OR "preoperative planning" OR "surgical instrumentation" OR "patient-specific models" OR "anatomic models" OR "medical devices")).

### Reference standard

The reference standard was a Pubmed (https://pubmed.ncbi.nlm.nih.gov/) search.

### Analysis of the response

When this search string was entered in Pubmed (pubmed.ncbi.nlm.nih.gov), it resulted in 105 articles mostly associated with the intended subject of 3D printing and arthroscopy. This was considered to be a valuable strategy to perform the search, and the hybrid use of ChatGPT plus the manual entry of its output into Pubmed was considered a far better strategy for future use.

## Query 10

### Typical person making the query and rationale:

This query was from an undergraduate medical 3D printing volunteer.

### Details of the query and complete response

#### Query

What are the basics of 3D printing in medicine?

#### Response

1. Medical Imaging: The process typically starts with medical imaging techniques such as computed tomography (CT) scans, magnetic resonance imaging (MRI), or 3D ultrasound. These imaging modalities help capture detailed anatomical data of a patient's specific body part or organ.
2. Digital Design: The imaging data is then converted into a digital model using specialized software. This step involves segmentation, which isolates the region of interest (e.g., an organ or a bone) from the rest of the image. The digital model serves as a blueprint for the 3D printing process.
3. 3D Printing Technologies: There are various 3D printing technologies used in medicine, including stereolithography (SLA), selective laser sintering (SLS), fused deposition modeling (FDM), and polyjet printing. Each technology has its advantages and is chosen based on the specific requirements of the printed object.
4. Materials: Different materials can be used for 3D printing in medicine, including biocompatible polymers, metals, ceramics, and hydrogels. The choice of material depends on the application and the desired properties of the final printed object.
5. Surgical Planning and Training: 3D-printed anatomical models can be used for surgical planning. Surgeons can examine and practice complex procedures on accurate patient-specific replicas before performing the actual surgery, improving precision and reducing surgical risks.
6. Custom Implants and Prosthetics: 3D printing enables the production of patient-specific implants, prosthetics, and orthotics. Using patient imaging data, custom implants can be designed and printed to fit precisely, improving patient outcomes and comfort.
7. Medical Education and Research: 3D-printed models can be used for medical education, allowing students and healthcare professionals to gain hands-on experience with realistic anatomical structures. They can also be utilized for research purposes, enabling the study of diseases, testing new treatments, and advancing medical innovation.
8. Bioprinting: While still in the early stages of development, bioprinting involves the fabrication of living tissues and organs using bioinks, which consist of living cells. Bioprinting holds great potential for regenerative medicine, tissue engineering, and the development of patient-specific organ replacements.

### Reference standard

Reference standard was discussion with a radiologist who manages a 3D printing clinical program in an HCF.

### Analysis

The response was accurate and practically useful for a trainee working in an HCF 3D Printing lab. Based on this success, the following additional queries were made to ChatGPT.

## Query 11

### Typical person making the query and rationale:

This follow-up query was from also from an undergraduate medical 3D printing volunteer.

### Details of the query and complete response

#### Query

I am an undergraduate looking to learn 3D printing in medicine. What must I know to succeed?

#### Response

1. Basics of 3D Printing: Familiarize yourself with the fundamentals of 3D printing, including different technologies, materials, and processes involved. Understand the strengths and limitations of each technology and its applications in the medical field.
2. Medical Imaging: Gain a solid understanding of medical imaging techniques such as CT scans, MRI, or ultrasound. Learn how to interpret imaging data and extract relevant anatomical structures for 3D printing.
3. 3D Design Software: Acquire proficiency in 3D design software used for creating and manipulating digital models. Popular software options include Autodesk Fusion 360, Rhino3D, or Blender. Learn how to perform segmentation and generate accurate digital models from medical imaging data.
4. Anatomical Knowledge: Develop a strong foundation in human anatomy. Understand the structure and function of various organs, bones, and anatomical systems to accurately interpret medical images and design anatomically precise models.
5. CAD Modeling: Master computer-aided design (CAD) techniques and principles. Learn how to create accurate and functional 3D models, optimize designs for 3D printing, and ensure proper support structures and tolerances.
6. 3D Printing Technologies: Explore different 3D printing technologies used in medicine, including their capabilities, advantages, and limitations. Understand the specific requirements for printing medical-grade objects and the implications of different materials and printing processes.
7. Research and Collaboration: Stay updated with the latest research and developments in 3D printing in medicine. Engage in interdisciplinary collaborations with researchers, medical professionals, and engineers to gain practical insights and contribute to the field.
8. Hands-on Experience: Seek practical opportunities to work with 3D printers and gain hands-on experience in the entire 3D printing workflow, from imaging and model creation to post-processing and quality control. Consider internships, research projects, or extracurricular activities that provide exposure to medical 3D printing.
9. Ethics and Regulations: Familiarize yourself with ethical considerations, patient confidentiality, and regulatory aspects related to 3D printing in medicine. Understand the importance of patient consent, data privacy, and compliance with regulatory guidelines, such as those issued by the FDA or other relevant regulatory bodies.
10. Continuous Learning: Stay curious and committed to continuous learning in this rapidly evolving field. Follow industry trends, attend conferences, workshops, and webinars, and engage with professional networks or online communities dedicated to medical 3D printing.

### Reference standard

Reference standard was discussion with a radiologist who manages a 3D printing clinical program in an HCF.

### Analysis of the response

ChatGPT provided a road map similar to reference standard advice from several senior lab members, including the radiologist who manages the operations and research. Admittedly, the question posed was vague. However, segmentation was not discussed. One possible explanation is that segmentation is typically best taught in a “hands-on” fashion, and thus there is a relative paucity of text resources for ChatGPT to learn from and to provide instructions. Moreover, the software suggested did not include some common tools used in HCFs.

Based on these results, ChatGPT was asked an additional question that was specific to segmentation.

## Query 12

### Typical person making the query and rationale:

This follow-up query was also from an undergraduate medical 3D printing volunteer.

#### Query

How can I learn the process of segmentation of 3D models as an undergraduate student with minimal understanding of patient anatomy?

#### Response

1. Anatomy Fundamentals: Start by gaining a basic understanding of human anatomy. Utilize textbooks, online resources, or educational apps to familiarize yourself with anatomical structures. Focus on major organs, bones, and systems relevant to the medical field.
2. Online Courses and Tutorials: Look for online courses or tutorials that specifically cover medical image segmentation. Websites like Coursera, edX, and Udemy offer courses on medical imaging and segmentation. These courses can provide a structured learning experience and introduce you to the concepts and techniques used in segmentation.
3. Open-Source Software: Explore open-source software options that are commonly used for medical image segmentation, such as 3D Slicer, ITK-SNAP, or Simpleware ScanIP. These software tools often have user-friendly interfaces and provide tutorials or documentation to help beginners.
4. Practice with Sample Data: Look for publicly available sample medical imaging datasets. These datasets typically come with pre-segmented structures, allowing you to compare your segmentation results and learn from them. Practice segmenting different anatomical structures using the software tools mentioned earlier.
5. Collaborate with Medical Professionals: Reach out to medical professionals, such as radiologists or researchers working in medical imaging, and ask for guidance or collaboration opportunities. They can provide valuable insights, share real-world cases, and offer guidance on the segmentation process.
6. Join Online Communities: Engage with online communities and forums focused on medical imaging and 3D printing in medicine. Platforms like LinkedIn, Reddit, or specialized forums allow you to connect with professionals in the field who can provide advice, answer questions, and share resources.
7. Research Projects or Internships: Look for research projects or internships in academic institutions, hospitals, or companies involved in medical imaging or 3D printing in medicine. These opportunities can provide hands-on experience, mentorship, and exposure to real-world segmentation challenges.
8. Continued Learning and Practice: Keep up with the latest advancements in medical imaging and segmentation through journals, conferences, and research papers. Continuously practice segmentation on various datasets to improve your skills and gain confidence.

### Reference standard

Reference standard was discussion with a radiologist who manages a 3D printing clinical program in an HCF.

### Analysis of the response

ChatGPT provided an accurate overview, although tutorials do exist for software that is sold to HCFs as medical devices. Some of the information was considered very general, despite the narrow focus of this query.

## Summary and conclusions

ChatGPT demonstrated higher accuracy and usefulness when dealing with simpler medical 3D printing questions. However, as the complexity of the medical inquiries increased, ChatGPT's performance suffered. Specifically, for the most intricate questions, the accuracy of the responses declined.

Despite ChatGPT's inaccuracies, the language used by the models remained highly confident, and therefore was misleading. Tasks that involved imaging or physical model manipulation were particularly challenging for LLMs, and their ability to provide successful responses was limited in those cases. The lower performance of ChatGPT can be attributed to the fact that hands-on skills in medical 3D printing are typically acquired through mentorship and often involve "trial and error". Consequently, considering the current state of the software (June 2023), ChatGPT proved to be valuable for junior trainees who are actively involved in medical 3D printing labs within HCFs. However, it is important to note that the information provided by ChatGPT should not be seen as a substitute for mentorship and practical experience. As the field progresses towards more conventional medical practices, caution should be exercised when relying on ChatGPT, and its outputs should always be verified by a qualified healthcare professional.

There is an important, alternative way to interpret the findings – one that uses ChatGPT as a lens to assess the portfolio of educational information available for medical 3D printing. 3D printing is relatively new to HCFs, and the educational material is still limited; when medical devices are generated by industry and sold to providers and HCFs, there is less academic incentive to teach the broader healthcare sector. The bottom line is that there remains a large, unmet need for medical 3D printing educational prose at all levels: undergraduate, medical student, resident, biomedical engineer, and providers. ChatGPT should improve as educational materials become more readily available. For example, ChatGPT has been applied to the United States Medical Licensing Examination (USMLE) [30] and has been proven to pass the American Board of Radiology (ABR) specialty examinations [31]. For these educational tasks, there is voluminous material available.

While this editorial does not statistically test a hypothesis, it explores additional common themes relevant to medical 3D printing. First, generative AI 3D printing education is more valuable for generalities than it is for specifics. Due to limited educational opportunities, 3D printing in HCFs still relies heavily on mentorship. Moreover, learning the nuances of medical 3D printing always involves a significant "hands-on" component. These are suboptimally captured in a refined human survey. Generative AI exhibits specific limitations, such as exclusion of a discussion of image segmentation, possibly due to dominance of “non-medical” 3D printing literature as opposed to 3D printing for medical applications. Thus, medical image segmentation is statistically less favored to be selected as the desirable ChatGPT output. However, more specific queries yield improved responses.

Lastly, a direct literature search on 3D printing through ChatGPT had several notable deficiencies. Nevertheless, using ChatGPT to generate a Boolean search string for PubMed proved valuable, offering a workaround that may be adopted to refine ChatGPT prompts used in other medical fields.

## References

[1] Masters K (2023). Ethical use of artificial intelligence in health professions education: AMEE guide no. 158. Med Teach..

[2] Ötleş E, James CA, Lomis KD, Woolliscroft JO. Teaching artificial intelligence as a fundamental toolset of medicine. Cell Rep Med. 2022;3:100824. 10.1016/j.xcrm.2022.100824 PMC9797941 36543111

[3] Tolsgaard MG, Pusic MV, Sebok-Syer SS, Gin B, Svendsen MB, Syer MD, et al. The fundamentals of Artificial Intelligence in medical education research: AMEE Guide No. 156. Med Teach. 2023;45:565–73. 10.1080/0142159X.2023.2180340 36862064

[4] Biswas S. ChatGPT and the Future of Medical Writing. Radiology. 2023;307:e223312. 10.1148/radiol.223312 36728748

[5] Dave T, Athaluri SA, Singh S. ChatGPT in medicine: an overview of its applications, advantages, limitations, future prospects, and ethical considerations. Front Artif Intell. 2023;6:1169595. 10.3389/frai.2023.1169595 PMC10192861 37215063

[6] Gordijn B, Have HT. ChatGPT: evolution or revolution? Med Health Care Philos. 2023;26:1–2. 10.1007/s11019-023-10136-0 36656495

[7] Luitse D, Denkena W (2021). The great Transformer: Examining the role of large language models in the political economy of AI. Big Data Soc.

[8] Brown T, Mann B, Ryder N, Subbiah M, Kaplan JD, Dhariwal P, et al. Language models are few-shot learners. Adv Neural Inf Process Syst. 2020;33:1877–901.

[9] Chow JCL, Sanders L, Li K. Impact of ChatGPT on medical chatbots as a disruptive technology. Front Artif Intell. 2023;6:1166014. 10.3389/frai.2023.1166014 PMC10113434 37091303

[10] Ray TR, Kellogg RT, Fargen KM, Hui F, Vargas J. The perils and promises of generative artificial intelligence in neurointerventional surgery. J Neurointerv Surg. 2023. Advance online publication. 10.1136/jnis-2023-020353. 10.1136/jnis-2023-020353 37438101

[11] Lecler A, Duron L, Soyer P (2023). Revolutionizing radiology with GPT-based models: current applications, future possibilities and limitations of Chat GPT. Diagn Interv Imaging.

[12] Rahsepar AA, Tavakoli N, Kim GHJ, Hassani C, Abtin F, Bedayat A. How AI Responds to Common Lung Cancer Questions: ChatGPT vs Google Bard. Radiology. 2023;307:e230922. 10.1148/radiol.230922 37310252

[13] Haver HL, Lin CT, Sirajuddin A, Yi PH, Jeudy J. Use of ChatGPT, GPT-4, and Bard to Improve Readability of ChatGPT's Answers to Common Questions on Lung Cancer and Lung Cancer Screening. AJR Am J Roentgenol. 2023. Advance online publication. 10.2214/AJR.23.29622. 10.2214/AJR.23.29622 37341179

[14] Sirjani E, Cragg PJ, Dymond MK. Glass transition temperatures, melting temperatures, water contact angles and dimensional precision of simple fused deposition model 3D prints and 3D printed channels constructed from a range of commercially available filaments. Chem Data Collect. 2019;22:100244.

[15] Cacace S, Cristiani E, Rocchi L. A level set based method for fixing overhangs in 3D printing. Appl Math Model. 2017;44:446–55.

[16] Fedorov A, Beichel R, Kalpathy-Cramer J, Finet J, Fillion-Robin JC, Pujol S, et al. 3D Slicer as an image computing platform for the Quantitative Imaging Network. J Magn Reson. 2012;30:1323–41. 10.1016/j.mri.2012.05.001 PMC3466397 22770690

[17] Mitsouras D, Liacouras PC, Wake N, Rybicki FJ. RadioGraphics Update: Medical 3D Printing for the Radiologist. Radiographics. 2020;40:E21–3. 10.1148/rg.2020190217 32609597

[18] Chen D, Ganapathy A, Abraham N, Marquis KM, Bishop GL, Rybicki FJ, et al. 3D printing exposure and perception in radiology residency: survey results of radiology chief residents. 3D Print Med. 2023;9:13. 10.1186/s41205-023-00173-z PMC10133904 37103761

[19] Garcia J, Yang Z, Mongrain R, Leask RL, Lachapelle K. 3D printing materials and their use in medical education: a review of current technology and trends for the future. BMJ Simul Technol Enhanc Learn. 2018;4:27–40. 10.1136/bmjstel-2017-000234 PMC5765850 29354281

[20] Introduction to Medical 3D Printing: What is actually possible? https://www.sculpteo.com/en/3d-learninghub/basics-of-3d-printing/what-is-3d-printing/. Accessed 20 May 2023.

[21] Free readability formulas. free readability tools : readability calculators. https://readabilityformulas.com/. Accessed 20 May 2023.

[22] Azamfirei R, Kudchadkar SR, Fackler J. Large language models and the perils of their hallucinations. Crit Care. 2023;27:1–2. 10.1186/s13054-023-04393-x PMC10032023 36945051

[23] Tack P, Victor J, Gemmel P, Annemans L. 3D-printing techniques in a medical setting: a systematic literature review. Biomed Eng Online. 2016;15:1–21. 10.1186/s12938-016-0236-4 PMC5073919 27769304

[24] Wong KC (2016). 3D-printed patient-specific applications in orthopedics. Orthop Res Rev.

[25] Li H, Fan W, Zhu X (2020). Three-dimensional printing: The potential technology widely used in medical fields. J Biomed Mater Res A.

[26] Crafts TD, Ellsperman SE, Wannemuehler TJ, Bellicchi TD, Shipchandler TZ, Mantravadi AV. Three-Dimensional Printing and Its Applications in Otorhinolaryngology-Head and Neck Surgery. Otolaryngol Head Neck Surg. 2017;156:999–1010. 10.1177/0194599816678372 28421875

[27] Vukicevic M, Mosadegh B, Min JK, Little SH. Cardiac 3D Printing and its Future Directions. JACC Cardiovasc Imaging. 2017;10:171–84. 10.1016/j.jcmg.2016.12.001 PMC5664227 28183437

[28] Lin M, Firoozi N, Tsai CT, Wallace MB, Kang Y. 3D-printed flexible polymer stents for potential applications in inoperable esophageal malignancies. Acta Biomater. 2019;83:119–29. 10.1016/j.actbio.2018.10.035 30366130

[29] Yang WF, Choi WS, Leung YY, Curtin JP, Du R, Zhang CY, Chen XS, Su YX. Three-dimensional printing of patient-specific surgical plates in head and neck reconstruction: A prospective pilot study. Oral Oncol. 2018;78:31–6. 10.1016/j.oraloncology.2018.01.005 29496055

[30] Kung TH, Cheatham M, Medenilla A, Sillos C, De Leon L, Elepaño C, et al. Performance of ChatGPT on USMLE: Potential for AI-assisted medical education using large language models. PLoS Digit Health. 2023;2:e0000198. 10.1371/journal.pdig.0000198 PMC9931230 36812645

[31] Bhayana R, Krishna S, Bleakney RR. Performance of ChatGPT on a radiology board-style examination: Insights into current strengths and limitations. Radiology. 2023;307:e230582. 10.1148/radiol.230582 37191485

